# Multiorgan Failure and Omicron: A Suspected Case of Post-COVID-19 Cholangiopathy

**DOI:** 10.7759/cureus.35010

**Published:** 2023-02-15

**Authors:** Ricardo Anguiano-Albarran, Daniel Cain, Mohammad Ashfaq, Apurva Modi, Shovendra Gautam

**Affiliations:** 1 Internal Medicine, Texas Christian University School of Medicine – Internal Medicine Residency Program, Fort Worth, USA; 2 Transplant Hepatology, Baylor Simmons Transplant Institute, Baylor Scott & White All Saints Medical Center, Fort Worth, USA; 3 Graduate Medical Education, Texas Christian University School of Medicine – Internal Medicine Residency Program, Fort Worth, USA

**Keywords:** gastroenterology, ssc, ssc-cip, covid cholangiopathy, coronavirus, hepatology, cholangiopathy, covid-19

## Abstract

Since the declaration of a global pandemic by the World Health Organization on March 11, 2020, coronavirus disease 2019 (COVID-19) has impacted millions worldwide. This complex disease process has been primarily associated with respiratory illness. As we continue to learn about COVID-19, there appears to be a growing spectrum of non-pulmonary manifestations. A major topic of interest is hepatic dysfunction related to COVID-19, specifically the growing number of cases involving acute liver failure in the setting of COVID-19. Here, we present a rare case of a patient with COVID-19 antibodies, negative inpatient COVID-19 testing, jaundice, and elusive multiorgan dysfunction.

## Introduction

The coronavirus disease 2019 (COVID-19) has impacted millions of people worldwide since the declaration of the pandemic by the World Health Organization [1]. The breadth of organ systems impacted in those infected continues to be a point of study. Laboratory abnormalities in hepatic function are routinely identified in COVID-19 patients. The exact pathophysiology of hepatic injury related to COVID-19 is not yet fully understood. Some of the leading hypotheses include direct hepatic injury through angiotensin-converting enzyme II (ACE-2) receptor hijacking, cytokinetic immune system dysregulation, and increased drug metabolite byproducts such as reactive oxygen species (ROS) [2,3]. Currently, the ACE-2 receptor remains one of the major foci of research in comprehending the multiorgan involvement of coronavirus. It is generally accepted that ACE-2 receptors serve as a key viral entry point in pulmonary tissue [2,3]. Research has identified the presence of ACE-2 receptors in cholangiocytes [4]. To date, no significant presence has been identified in hepatocytes [5]. Therefore, it is postulated that one of the mechanisms of hepatic injury may be through a sclerosing-type mechanism. Identified as secondary sclerosing cholangitis in critically ill patients (SSC-CIP), this rare phenomenon can manifest as acute liver dysfunction. As documented by Durazo et al., a case of SSC-CIP was ultimately treated with orthotopic liver transplantation [6]. Within the spectrum of SSC-CIP is COVID-19-associated cholangiopathy [7]. Recent data suggest that COVID-19 may be a potential cause of secondary sclerosing cholangitis [7]. This rare presentation is postulated to be due to an uncontrolled systemic inflammatory response in conjunction with ischemic injury. This multiple-hit injury mechanism then leads to the induction of biliary sclerotic changes and subsequent hepatic dysfunction.

A multiple-hit hypothesis proposes that initial hepatic injury may be further propagated by the production of oxidative metabolites during an inappropriate and exaggerated immune response. Termed cytokine storm, this acute, pro-inflammatory state has been observed in critically ill COVID-19 patients. During a cytokine storm state, the immune system produces inflammatory signals by way of interleukins, tumor necrosis factor, and several other chemokines. This unchecked response ultimately increases vascular permeability and immune cell recruitment [8,9]. The recruitment of immune cells, particularly neutrophils, sets the stage for a pro-oxidative reactive state. It is well-established that neutrophils can release myeloperoxidase (MPO). MPO is a heme enzyme that demonstrates P450-like activity through unique reactive mechanisms that include peroxidase activity, production of oxidative equivalents, and superoxide radical-catalyzed hydroxylation [10]. A key and noteworthy difference between MPO and P450 activity is the uncaging of MPO reactive byproducts, such as ROS. During P450-mediated drug metabolism, potential ROS enzyme-substrate complexes are caged to prevent radical diffusion to the surrounding medium [11]. Furthermore, MPO can propagate signaling mechanisms that result in the production of ROS such as hypochlorous acid (HOCI), superoxide (O_2_^•-^), and hydrogen peroxide (H_2_O_2_) [9,12]. Organ systems, such as hepatic injury in this case, may suffer severe cytotoxic tissue injury from the excess concentration of ROS resulting in DNA strand breakage and increased inflammatory response. ROS has been studied and attributed to further cellular damage in the setting of COVID-19 [13].

## Case presentation

A 68-year-old female had a pertinent medical history which included rheumatoid arthritis, atrial fibrillation on chronic anticoagulation, deep venous thrombosis, and hypertension. Notably, there was no known history of chronic liver disease (CLD) in the patient or her family. She presented with acute-onset jaundice. About 10 days prior to the presentation, the patient had experienced flu-like symptoms. The patient reportedly tested positive for COVID-19 at an urgent care clinic and was prescribed a course of doxycycline. The authors of this paper do not have access to the urgent care records and are unable to verify why antibiotic therapy was initiated. On the day prior to admission, only one 100 mg tablet of doxycycline had been ingested. Her other home medications included apixaban 5 mg, levothyroxine 200 mg, prednisone 5 mg, carvedilol 3.125 mg, aspirin 81 mg, ergocalciferol, 1,250 µg, and calcium supplements. She had not received biologic therapy within 12 months of her admission. The body mass index (BMI) was 33.93 kg/m^2^. The patient was not vaccinated against COVID-19. Upon initial evaluation, the patient was encephalopathic, diffusely jaundiced with scleral icterus, in atrial fibrillation with a rapid ventricular response, and tachypnea with decreased breath sounds in bilateral lower lung fields. Pertinent lab findings are included in Table 1 and were concerning for acute renal and liver failure.

**Table 1:** Notable lab findings during the beginning of the patient’s admission.

Lab	Result	Reference range
White blood cell count	15.9 K/µL	3.8–11.8 K/µL
Hemoglobin	11.2 g/dL	11.5–15.5 g/dL
Hematocrit	33.70%	35–45%
Platelet	421K/µL	130–400 K/µL
International normalized ratio	2.4	
Prothrombin time	29.1 seconds	9–12 seconds
Activated partial thromboplastin time	27.9 seconds	25.1–36.5 seconds
Ferritin	3149 ng/mL	9–150 ng/mL
C-reactive protein	12.7 mg/dL	0–0.3 mg/dL
Blood urea nitrogen	126 mg/dL	8–23 mg/dL
Serum creatinine	5.54 mg/dL	0.50–0.90 mg/dL
Total bilirubin	24.9 mg/dL	0–1.2 mg/dL
Direct bilirubin	14.4 mg/dL	0–0.4 mg/dL
Aspartate aminotransferase	525 IU/L	0–40 IU/L
Alanine aminotransferase	583 IU/L	0–32 IU/L
Alkaline phosphatase	201 U/L	44–121 IU/L
Albumin	1.3 g/dL	3.8–4.8 g/dL
Lactic acid	3.4 mmol/L	0.9–1.7 mmol/L

Due to acute liver failure, hepatorenal syndrome was initially considered but could not be properly evaluated in the setting of acute infection. Superimposed COVID-19 nephropathy with concomitant prerenal azotemia secondary to dehydration was the leading diagnosis due to the fractional excretion of sodium of 0.96% and improvement with hydration. Her urinalysis was also significant for amber color, proteinuria, bilirubin, and urobilinogen all being elevated. This also served as evidence of hepatic involvement. A combination of intravenous albumin, midodrine, and octreotide was administered on the first three inpatient days. With aggressive fluid resuscitation and increased oral intake of fluids, partial renal recovery was noted with downtrends in serum creatinine levels and adequate daily urine output. Fortunately, dialysis was not deemed necessary due to improved renal function.

Leukocytosis was thought to be related to ongoing multiorgan dysfunction and a pro-inflammatory state from COVID-19. Atrial fibrillation with rapid ventricular response was noted on telemetry upon admission. The patient was started on a diltiazem drip and transitioned to oral beta-blockade for rhythm maintenance, which she tolerated well. It is important to note that the patient remained normotensive throughout the entire hospital course. Elevated lactic acid was suspected to be due to ongoing hepatic dysfunction. Broad-spectrum antibiotic therapy was initiated but ultimately discontinued as there was no clear infectious source. There were no pathogens identified on blood cultures, and urine studies were negative for an underlying urinary tract infection. A chest X-ray completed upon admission demonstrated bilateral opacities compatible with COVID-19 pneumonia. No consolidations or effusions that would suggest a bacterial source were identified. Interestingly, the patient tested negative for COVID-19 twice while at our facility. A follow-up IgG severe acute respiratory syndrome coronavirus 2 (SARS-CoV-2) antibody assay returned positive. These results supported a timeline of COVID-19 illness within the last 10-14 days. Despite antibody positivity, there was well-documented evidence of breakthrough infection from the Omicron variant [14]. This is significant as she was exhibiting breakthrough COVID-19 infection in the presence of antibodies. Intravenous dexamethasone therapy was initiated. A total of eight days of daily 6 mg IV dexamethasone were completed. Importantly, the patient’s supplemental oxygen requirements were minimal during her admission, requiring only 2-3 L on a nasal cannula to maintain saturation above 92%. Monoclonal antibody therapy was deferred due to multiorgan dysfunction. Remdesivir was also not initiated due to the patient being outside the effective treatment window. The patient demonstrated a positive response to steroid therapy and was successfully weaned off supplemental oxygen.

**Figure 1:**
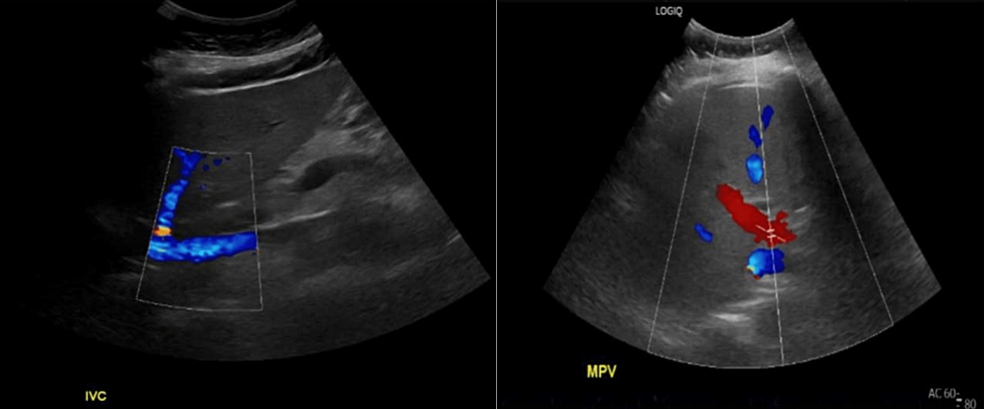
Doppler abdominal ultrasound demonstrating inferior vena cava (left) and main portal vein patency (right).

A broad serologic workup for acute hepatic disease was completed (Table 2). This serologic workup was, for the most part, unremarkable. Ischemic and drug-induced liver injury were high on the differential until further workup ruled them out. There was no recent history of recurrent thrombotic events while the patient was on anticoagulation therapy for atrial fibrillation. Nonetheless, a Doppler ultrasound of the abdomen was completed. This study found no acute vascular compromise to the hepatic vascular network (Figure 1). Over the course of her admission, her liver and renal functions improved and she was followed up on an outpatient basis for follow-up and treatment of non-alcoholic fatty liver disease (NAFLD).

**Table 2:** Serologic evaluation of abnormal hepatic function. SARS-CoV-2: severe acute respiratory syndrome coronavirus 2; NAA: nucleic acid amplification; NAAT: nucleic acid amplification test; PCR: polymerase chain reaction

Serologic testing	Result
SARS-CoV-2, NAA	Not detected (×2)
SARS-CoV-2 nucleocapsid antibody	IgG positive
Acetaminophen level	<3.0 µg/mL
Phosphatidyl ethanol level	<10 ng/dL
Hepatitis B surface antigen	Negative
Hepatitis B core IgM	Negative
Hepatitis A IgM antibody	Negative
Hepatitis C antibody	Negative
Hepatitis C quantitative NAAT	Negative
Anti-mitochondrial antibody	4.4 U; negative
Smooth muscle antibody	36 U; weak positive
Smooth muscle antibody titer, IgG	<1:20
Liver-kidney microsome antibody, IgG	<1:20
Herpes simplex virus PCR	Not detected
Ceruloplasmin	56 mg/dL; normal
Alpha-1-antitrypsin antibody	Negative
Immunoglobulins	IgA 718; IgG 1,069
Cytomegalovirus viral load, PCR	Not detected
Epstein-Barr virus, PCR	Detected, viral load 661 IU/mL

## Discussion

Ischemic liver injury was ultimately ruled out for three main reasons. First, the patient had no history of heart failure. An echocardiogram showed normal ejection fraction and right ventricular systolic pressure. With normal cardiac function, one would not expect underlying congestive hepatopathy. Second, blood pressure remained normotensive throughout the entire hospital course. Finally, Doppler imaging revealed no thrombotic burden.

**Figure 2:**
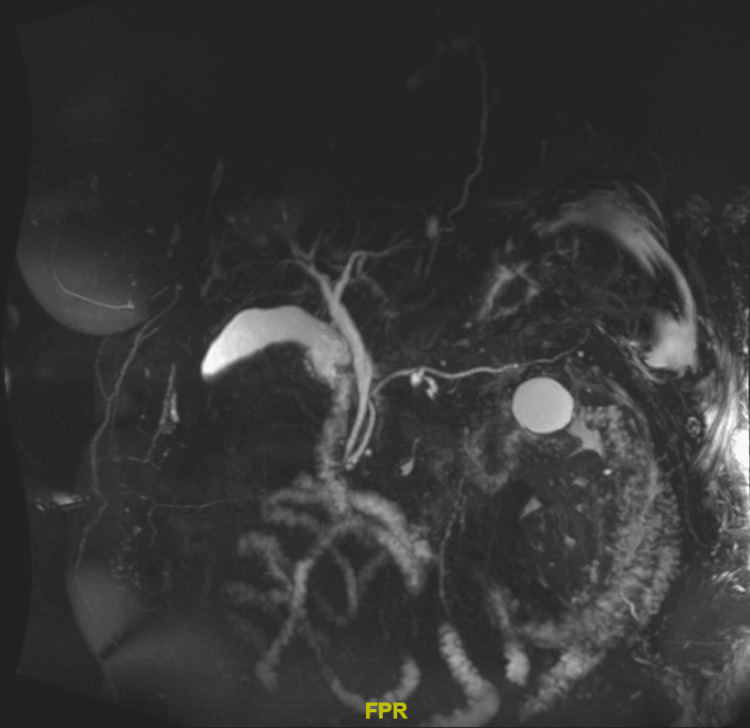
Magnetic resonance cholangiopancreatography demonstrating hepatic steatosis. No strictures or additional evidence of obstruction can be observed.

As mentioned previously, the patient reported taking only one 100 mg tablet of doxycycline. There are case reports documenting hepatoxicity from doxycycline use, but these occurrences are rare. Varma et al. reported a case of doxycycline-related cholestatic injury. In that case, the patient was exposed to 100 mg of doxycycline twice daily for three days [15]. Further evidence has identified doxycycline, when compared to other tetracyclines, as a safer alternative. Heaton et al. found that current or past users of doxycycline did not have an increased risk of developing hepatotoxicity [16]. With only one reported tablet consumed, we strongly believed doxycycline was not the root cause of liver injury. Epstein-Barr virus testing was positive. However, this was deemed inconsequential as the patient was likely a carrier of the virus. Biliary obstruction was ruled out through magnetic resonance cholangiopancreatography (Figure 2). This imaging study demonstrated a preserved hepatic contour, diffuse hepatic steatosis, and no evidence of biliary obstruction or choledocholithiasis.

In March 2021, the American Association for Study of Liver Diseases (AASLD) published a comprehensive statement for providers caring for liver patients during the COVID-19 pandemic. One interesting point mentioned associated risk factors for severe COVID-19 illness [17,18]. Data up until that time suggested that patients with NAFLD could be at a higher risk of severe COVID-19 infection [18]. Recently, the AASLD published a literature review related to COVID cholangiopathy. Metabolic syndrome and underlying CLD were noted as major risk factors associated with COVID-19 cholangiopathy [7]. Hartl et al. highlighted a cohort of 10 patients with underlying CLD who developed COVID-19 cholangiopathy. Of these 10 patients, seven had a background history of NAFLD. About 50% of the cohort, unfortunately, passed away, and one was evaluated for liver transplantation [19]. Due to the limited study population, it is hard to extrapolate these findings to guide clinical practice, but it cannot be ruled out that COVID-19 infection with underlying NAFLD may cause exacerbation of the underlying condition.

In brief, our patient was extensively evaluated for an etiology of acute hepatic failure. It is possible that the patient deteriorated due to the multi-hit hypothesis. At baseline, the patient had an established elevated risk profile due to underlying NAFLD and a BMI of 33 kg/m^2^. We propose the possibility of a rare instance of post-COVID-19 cholangiopathy. However, without a liver biopsy to demonstrate biliary sclerotic changes, this diagnosis remains debatable. Liver biopsy was strongly considered but ultimately deferred due to the patient’s clinical improvement and potential risks associated with an invasive procedure. Given that COVID-19 cholangiopathy can present as a mixed cholestatic and/or hepatocellular picture, our hypothesized diagnosis cannot be ruled out [6]. Our patient had elevated aspartate aminotransferase, alanine aminotransferase, alkaline phosphatase, bilirubin, and uric acid indicative of liver injury, in the absence of liver ischemia visualized on imaging. We hope that this case can further add to the literature for the continued understanding of COVID-19.

## Conclusions

Almost three years into the pandemic, we are still learning about the novel coronavirus. This case report may serve as evidence of the extrapulmonary manifestations of COVID-19, specifically regarding hepatobiliary involvement and the role underlying liver disease may have in patient presentations. The authors hope that this report guides readers to consider the other systems affected when evaluating COVID-19 patients. Further research is needed to grasp an in-depth understanding of hepatobiliary involvement in the setting of COVID-19.
